# Resistance monitoring and cross-resistance role of *CYP6CW1* between buprofezin and pymetrozine in field populations of *Laodelphax striatellus* (Fallén)

**DOI:** 10.1038/s41598-017-15294-9

**Published:** 2017-11-07

**Authors:** Yueliang Zhang, Yangchun Han, Baosheng Liu, Qiong Yang, Huifang Guo, Zewen Liu, Lihua Wang, Jichao Fang

**Affiliations:** 1 Institute of Plant Protection, Jiangsu Academy of Agricultural Sciences, Key Lab of Food Quality and Safety of Jiangsu Province-State Key Laboratory Breeding Base, Nanjing, 210014 China; 2 Key Laboratory of Monitoring and Management of Plant Disease and Insects, Ministry of Agriculture, Nanjing Agricultural University, Nanjing, China

## Abstract

Monitoring resistance and investigating insecticide resistance mechanisms are necessary for controlling the small brown planthopper, *Laodelphax striatellus*. The susceptibility to four common insecticides of *L*. *striatellus* collected from Jiangsu, Anhui, Zhejiang and Jilin provinces of China in 2015 was monitored. The results showed that all field populations remained susceptible to chlorpyrifos and thiamethoxam with resistance ratios (RRs) of 2.3- to 9.5 and 1.6- to 3.3, respectively, while the insects had developed moderate pymetrozine resistance with RRs of 18.7 to 34.5. Resistance against buprofezin had developed to an alarmingly high level in three southeastern provinces of China with RRs of 108.8 to 156.1, but in Jilin it had an RR of only 26.6. Moreover, in line with both the buprofezin and pymetrozine resistance levels, we found *LsCYP6CW1* to be over-expressed in all field *L*. *striatellus* populations, which indicated that it might be important for cross-resistance between buprofezin and pymetrozine. RNA interference (RNAi) ingestion resulted in the effective suppression of *LsCYP6CW1* expression, and significantly increased susceptibility to both buprofezin and pymetrozine compared with the control, which further confirmed that overexpression of *LsCYP6CW1* was involved in the cross-resistance to buprofezin and pymetrozine in field *L*. *Striatellus* populations.

## Introduction

The small brown planthopper (*Laodelphax striatellus* Fallén) (Homoptera: Delphacidae) is a notorious agricultural pest with a wide distribution range from Southeast Asia to Siberia and Europe that attacks several important agricultural crops including rice, corn, wheat, oat and barley^1^. Damage is inflicted not only by direct feeding but also by transmitting several plant viruses^2^. In China, this pest has been found in all rice-growing areas and has been a serious problem since 1999. In recent years, the outbreak frequency of this insect increased greatly^3^. It is believed that insecticide resistance may be the main contributor to such a population surge, as the control of this species mainly depends on applications of chemical insecticides^4^. Therefore, to better provide guidelines for the scientific use of pesticides in the resistance management of field *L*. *striatellus*, monitoring resistance to common insecticides and clarifying the development of resistance is necessary.

In China, the main insecticides used to control the small brown planthopper include pymetrozine, chlorpyrifos, buprofezin and thiamethoxam, and multiple reports indicate that *L*. *striatellus* has developed resistance to chlorpyrifos, buprofezin and pymetrozine^5,6^. The increasing resistance of rice planthopper to these insecticides was thought to be related to the widespread use of these insecticides to control *L*. *striatellus*. However, other possible factors might also be important, including shared detoxification resistance among different insecticides.

Increases in metabolism mediated by detoxification enzymes, such as cytochrome P450s, esterases and glutathione S-transferase, have been considered important for insecticide resistance^7,8^. Among the three detoxification enzymes, cytochrome P450s appear to be the major type of detoxification enzyme and are extensively involved in different types of insecticide resistance^9–11^. Cytochrome P450s constitute a large enzyme family found in almost all living organisms from bacteria to humans^9^. Insect P450s can metabolize diverse endogenous and exogenous compounds, such as insecticides and plant allele chemicals, and have been divided into four major clades: CYP2, CYP3, CYP4, and mitochondrial P450s^12,13^. CYP6s belong to the CYP3 family and are extensively involved in the development of insecticide resistance. In insects, the overexpression of some *CYP6* genes has been associated with the increased metabolism of insecticides in resistant strains, including *Drosophila melanogaster* (*CYP6G1*), *Musca domestica* (*CYP6D1*), *Bemisia tabaci* (*CYP6CM1*), *Culex pipiens pallens* (*CYP6F1*), *Nilaparvata lugens* (*CYP6ER1*, *CYP6AY1*), *Locusta migratoria* (*CYP6F*) and *Aphis gossypii* (*CYP6A2*)^14–21^. Our previous study found that overexpressed *CYP6CW1* was closely associated with buprofezin resistance in a lab-selected resistant strain^22^, but whether or not a similar resistance mechanism occurs in field populations is unclear.

This study aimed to (1) evaluate the susceptibility of the small brown planthopper to four commonly used insecticides in seven different field populations; (2) analyse the synergistic roles of piperonyl butoxide (PBO) and conduct P450 gene expression analyses of *L*. *Striatellus* in seven resistant field populations and the susceptible YN population; and (3) investigate the cross-resistance functions of *LsCYP6CW1* between buprofezin and pymetrozine via RNA interference (RNAi). The results will help to understand the insecticide detoxification function of *LsCYP6CW1* and benefit resistance management.

## Results

### Susceptibility of *L*. *striatellus* to insecticides

The susceptibility of field *L*. *Striatellus* populations to various tested insecticides is reported in Table 1. The LC_50_ of chlorpyrifos ranged from 17.8 mg/L to 74.6 mg/L, with RRs (resistance ratios) ranging from 2.3- to 9.5-fold, which suggests that field populations of *L*. *Striatellus* have developed low and moderate resistance to chlorpyrifos; the LC_50_ of pymetrozine ranged from 173.6 mg/L to 319.7 mg/L, with RRs ranging from 18.7- to 34.5-fold and indicating a moderate level of resistance. The LC_50_ of buprofezin was from 35.8 mg/L to 210.8 mg/L, with RRs from 26.6- to 156.1-fold, and resistance occurred at moderate or extremely high levels. Finally, the LC_50_ of thiamethoxam ranged from 7.3 mg/L to 15.3 mg/L, and these results show thiamethoxam susceptibility in *L*. *striatellus* field populations.

**Table 1:** Resistance levels of field populations and synergistic effects of PBO on the toxicity of four insecticides to the field populations of *L*. *striatellus*

Population and treatment	Chlorpyrifos		Pymetrozine		Buprofezin		Thiamethoxam	
	LC_50_ (mg L^−1^) (95% CL)	RR/SR	LC_50_ (mg L^−1^) (95% CL)	RR/SR	LC_50_ (mg L^−1^) (95% CL)	RR/SR	LC_50_ (mg L^−1^) (95% CL)	RR/SR
NJ	32.1 (23.1–44.5)	4.09/0.41	228 (174–297)	24. 6/1.52	185 (136–251)	136/0.95	13.1 (10.1–16.9)	2.86/0.70
NJ + PBO	77.7 (54.3–111)		150 (103–185)		195 (159–238)		18.5 (11.2–30.5)	
LY	48.1 (34.6–66.9)	6.14/1.08	235 (174–316)	25.3/1.20	211 (155 –286)	156/1.62	14.8 (9.59–23.0)	3.25/0.97
LY + PBO	44.4 (34.5–57.2)		195 (147–258)		130 (93.5–181)		15.2 (3.65–24.4)	
JH	45.7 (29.6–70.6)	5.83/0.88	320 (251–406)	34.5/1.27	147 (117–186)	109/0.71	15.3 (9.66–24.1)	3.34/1.87
JH + PBO	52.2 (32.2–84.7)		252 (187–340)		208 (166-259)		8.16 (6.22–10.7)	
JY	74.6 (41.4–134)	9.52/0.78	317 (269–374)	34.2/1.86	147 (108–200))	109/1.50	11.4 (8.84–14.6)	2.49/0.73
JY + PBO	96.1 (60.9–152)		171 (133–219)		97.9 (71.5–134)		15.5 (8.81–21.3)	
CC	17.8 (11.4–26.8)	2.27/0.79	174 (132–228)	18.7/0.67	35.8 (29.3–43.8)	26.6/0.93	7.32 (6.14–8.72)	1.60/0.74
CC + PBO	22.5 (18.6–27.2)		258 (200–368)		38.6 (27.1–55.0)		9.90 (7.31–13.4))	
JX	43.1 (36.4–50.9)	5.49/0.37	216 (162–289)	23.3/1.11	196 (162–238)	145/0.69	8.64 (7.15–10.4)	1.89/1.27
JX + PBO	151 (61.3–274)		195 (142–256)		283 (169–474)		6.80 (5.43–8.53)	
LJ	54.8 (46.1–65.2)	6.99/1.24	204 (125–286)	22.0/1.26	170 (80.1–269)	126/1.50	10.5 (6.56–16. 7)	2.29/1.26
LJ + PBO	44.1 (34.3–56.6)		152 (104–223)		113 (80.2–160)		8.30 (6.31–10.5)	
Lab-YN	7.84 (3.01–17.9)	1.00/1.08	9.27 (5.28–17.6)	1.00/1.01	1.35 (0.74–3.64)	1.00/0.92	4.57 (1.17–11.2)	1.00/1.03
YN + PBO	7.24 (5.57–12.8)		9.17 (6.52–15.9)		1.46 (0.67–3.72)		4.42 (3.35–5.54)	

^a^RR (resistance ratio) = LC_50_ of field populations/LC_50_ of YN population.

^b^SR (synergism ratio) = LC_50_ of insecticide/LC_50_ of insecticide + synergist.

### Synergistic effect evaluated

The results showed that PBO had no significant synergistic effect on chlorpyrifos in most field populations except for LJ, where the synergistic rate was 1.24. However, PBO exerted a negative synergistic effect (synergistic rate 0.37) on chlorpyrifos in the JX population. A moderate synergistic effect of PBO on pymetrozine was found in six field populations but not in the relatively susceptible CC population, with the synergistic rate varying from 1.11 to 1.86. Surprisingly, only three field populations of LY, JY and LJ showed synergistic effects of PBO on buprofezin, with rates of 1.62-, 1.50- and 1.50- fold, respectively. The other four field populations showed no synergistic effect of PBO on buprofezin. In line with the result of buprofezin, thiamethoxam showed a synergistic effect of PBO in only some field populations, with a synergistic rate of 1.87 in JH, 1.27 in JX and 1.26 in LJ, in contrast to the absence of an effect in the other populations (Table 1).

### Screening of *L*. *striatellus* P450 genes associated with insecticide resistance in the field strain

**Figure 1:**
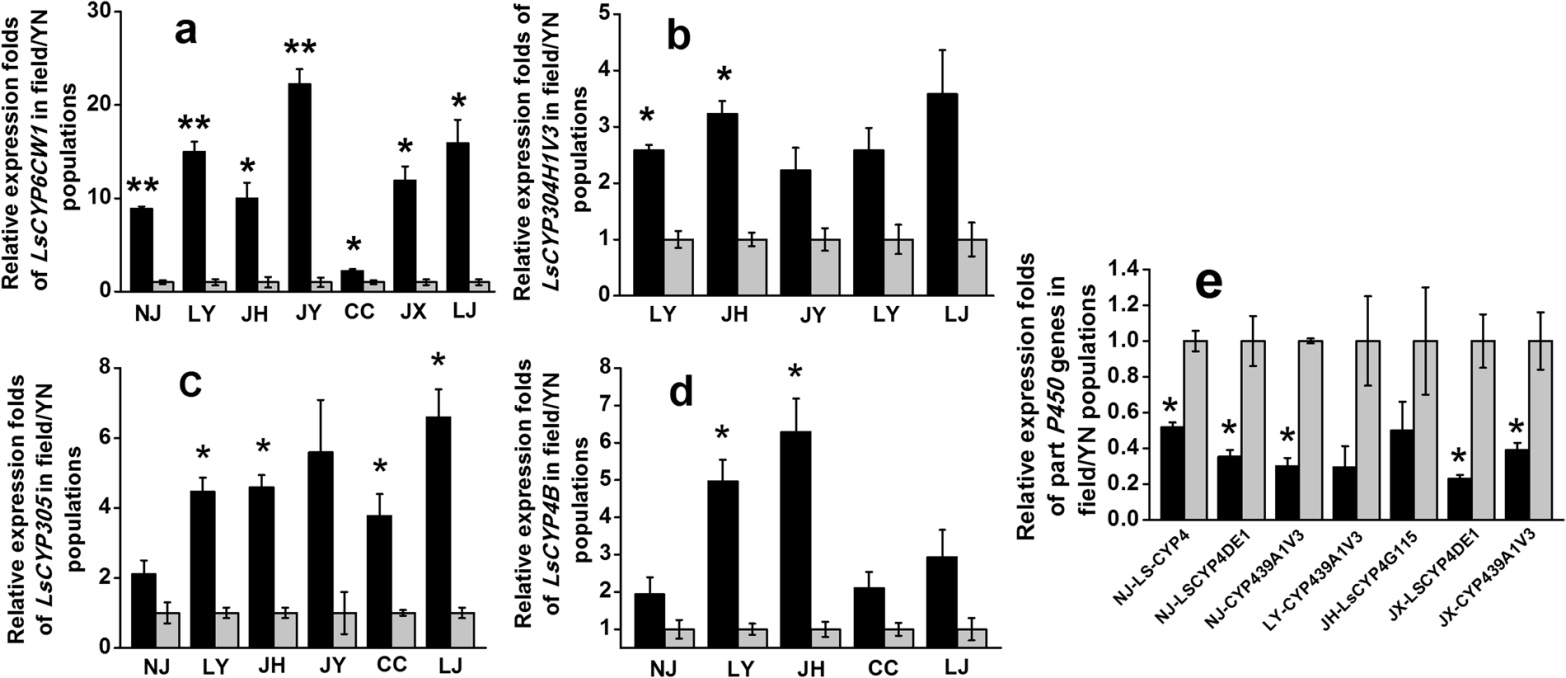
Relative expression fold changes of P450 genes in field populations (black represents NJ, LY, JH, JY, CC, JX and LJ) and the susceptible YN population (grey represents YN) of *L*. *striatellus*. Each bar indicates the mean of three biological samples, each of which involved 3 technical replicates. Error bars represent the standard deviation from the mean. Data were normalized to the expression of ADP ribosylation factor (ARF). The significantly different expression of P450 genes in the field populations compared with the control YN population (figure only shows expression differences of more than 2-fold) are marked by asterisks. *means significantly different at the 0.05 level and **means significantly different at the 0.01 level. (**a**) represents *LsCYP6CW1*, (**b**) represents *LsCYP304H1V3*, (**c**) represents *LsCYP305*, (**d**) represents *LsCYP4B* and (**e**) represents lower-expression genes in the field populations.

Of the 47 P450 genes of *L*. *striatellus*, *LsCYP6CW1* showed an 8.87-fold increase in expression for NJ, 14.96-fold for LY, 9.96-fold for JH, 22.24-fold for JY, 2.21-fold for CC, 11.92-fold for JX, and 16.92-fold for LJ, respectively, compared with the susceptible YN population (Fig. 1a). These expression levels of *LsCYP6CW1* were to some extent in accord with pymetrozine and buprofezin resistance levels in these field populations. Apart from *LsCYP6CW1*, other P450 genes also showed increased expression, including *LsCYP304H1V3*, which exhibited 2.58-fold higher expression for the LY population, 3.23-fold for JH, 2.23-fold for JY and 3.58-fold for LJ, respectively (Fig. 1b). *LsCYP305* showed 2.10-fold higher expression for NJ, 4.46-fold for LY, 4.59-fold for JH, 5.59-fold for JY, 3.37-fold for CC and 6.59-fold for the LJ population (Fig. 1c), respectively, and *LsCYP4B* showed 2.00-fold higher expression for NJ, 4.96-fold for LY, 6.28-fold for JH, 2.10-fold for CC and 2.93-fold for LJ (Fig. 1d). Furthermore, some P450 genes of *L*. *striatellus* showed lower expression in the field populations than in the YN population. These genes included *LsCYP4*, *LsCYP4DE1* and *LsCYP439A1V3*, which had 0.52-, 0.35- and 0.30-fold expression, respectively, for the NJ population. *LsCYP439A1V3* showed 0.29-fold expression for LY, *LsCYP4G115* had 0.5-fold expression for JH, and both *LsCYP4DE1* and *LsCYP439A1V3* had 0.23 and 0.39-fold lower expression, respectively, for JX (Fig. 1e).

### Knockdown of *LsCYP6CW1* increases sensitivity to both pymetrozine and buprofezin in field populations

**Figure 2:**
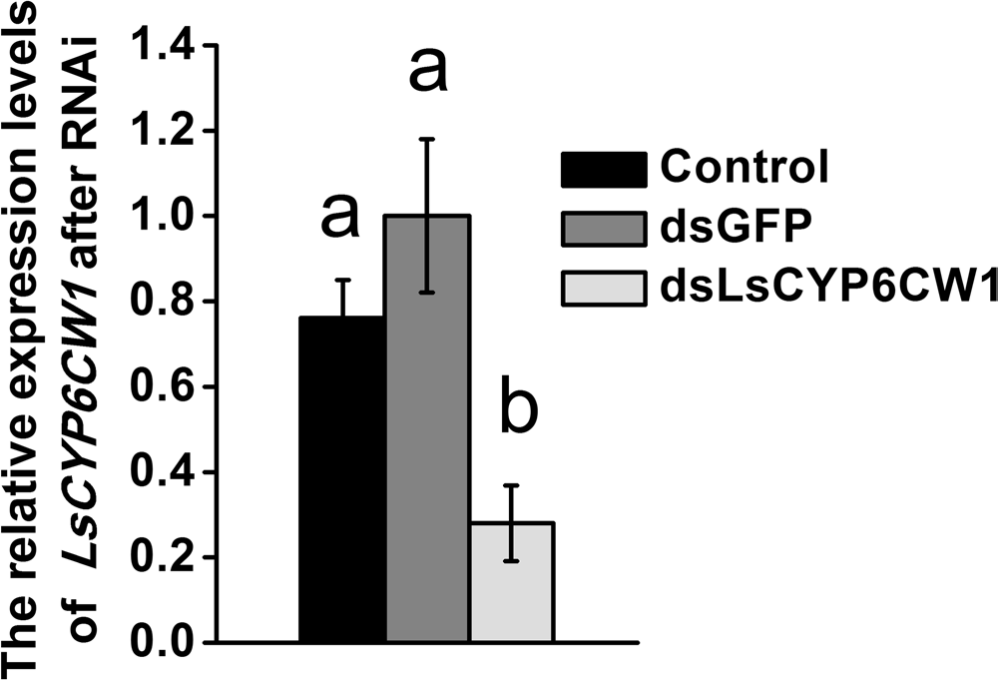
*LsCYP6CW1* mRNA expression levels after nymphs ingested artificial feed; dsGFP, dsRNA of green fluorescent protein (GFP); or ds*LsCYP6CW1*, dsRNA of *LsCYP6CW1*. The time from ingestion was 5 d, and the dose was 200 μg/mL. The levels of *LsCYP6CW1* transcription in *LsCYP6CW1* dsRNA-treated and untreated insects were normalized compared to dsGFP-treated individuals. Data are presented as the average of three biological replicates, each conducted in duplicate and normalized to a control gene with error bars representing SEM. Means depicted with different letters are significantly different by ANOVA(P < 0.05).

**Figure 3:**
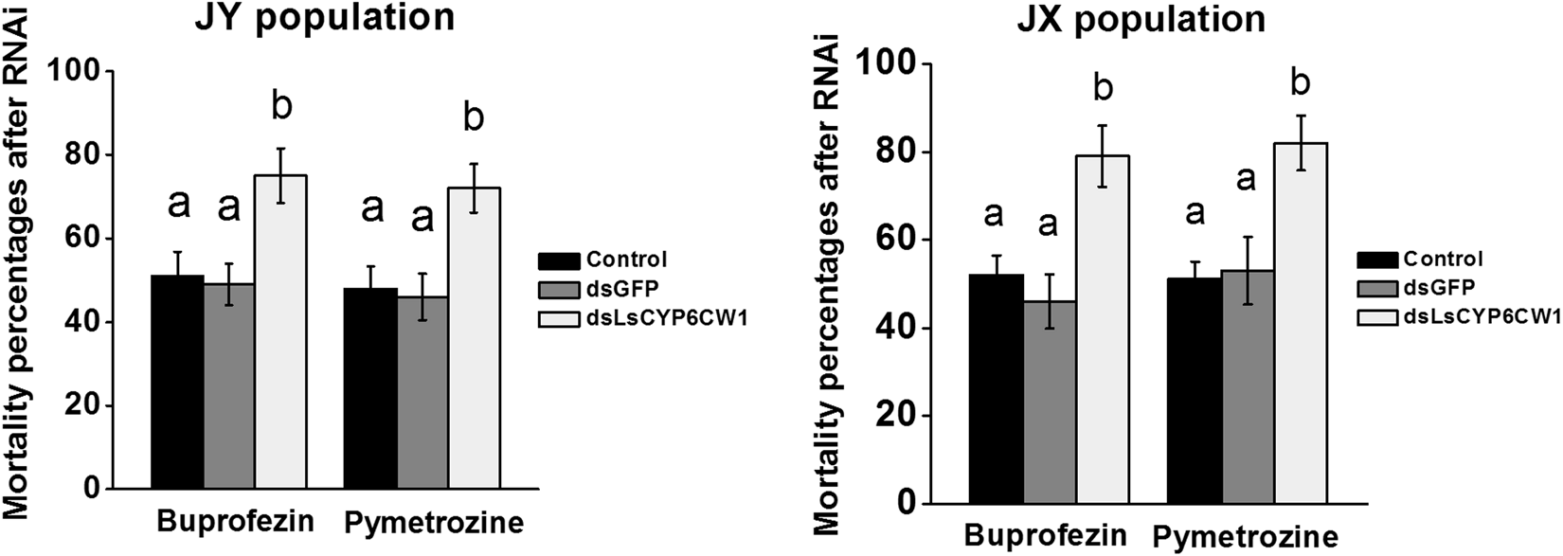
Mortality of *LsCYP6CW1*-silenced *L*. *striatellus* nymphs after buprofezin and pymetrozine treatment in the JY and JX populations, respectively. Control (nymphs that ingested artificial feed), *dsGFP* (nymphs that ingested dsRNA GFP), and *dsCYP6CW1* (nymphs that ingested dsRNA *LsCYP6CW1*). Data are presented as the average of three biological replicates, each conducted in duplicate and normalized to a control gene with error bars representing SEM. Means depicted with different letters are significantly different by ANOVA (P < 0.05).

*LsCYP6CW1* exhibited the highest expression in all field populations compared to the susceptible YN population, and the expression levels of *LsCYP6CW1* were consistent with the pymetrozine and buprofezin resistance levels. We therefore further evaluated the role of *LsCYP6CW1* in pymetrozine and buprofezin resistance via RNAi. Before conducting RNAi, the siRNA candidate target regions of *LsCYP6CW1* were identified through https://www.genscript.com/ssl-bin/app/rnai (Genscript Corporation). Moreover, previous studies found that RNAi ingestion could effectively suppress gene expression in small rice planthopper nymphs^23,24^. In this study, the 1st instar nymphs of *L*. *striatellus* were fed a dose of 0.2 mg/mL of *dsLsCYP6CW1* and *dsGFP*. The qPCR data showed that the *LsCYP6CW1* mRNA levels were dramatically decreased at 5 d. As shown in Fig. 2, the ingestion of 0.2 mg/mL of *dsLsCYP6CW1* for 5 d resulted in 0.28-fold lower expression than dsGFP ingestion and 0.37-fold lower expression than ingestion of an artificial diet only (control). LC_50_-discriminating doses of the JY and JX populations were used to investigate differences in susceptibility after RNAi. Knockdown of *LsCYP6CW1* in both the JY and JX populations significantly increased susceptibility to pymetrozine and buprofezin compared with the control; 75% and 72% died in the JY population and 79% and 82% died in the JX population following exposure to pymetrozine and buprofezin, respectively. These figures were significantly higher than those observed in the control groups, in which 49% and 46% of JY insects from the dsGFP treatment died following exposure to pymetrozine and buprofezin, respectively; equivalent figures for insects fed the artificial diet were 51% and 48%. For the JX population, 46% and 53% of insects died following exposure to pymetrozine and buprofezin, respectively, in the dsGFP treatment, and the equivalent figures were 52% and 51% when the insects were fed only an artificial diet (Fig. 3).

## Discussion

In order to maintain the long-term efficacy of insecticides, it is necessary to periodically assess the susceptibility levels and cross-resistance profiles of agricultural pests to the insecticides. In this study, the monitored field populations of *L*. *Striatellus* came mainly from eastern China, such as Jiangsu, Zhejiang and Anhui provinces, and these regions have developed higher levels of resistance than other regions in China^5,6,25^. One field population came from Jilin, in the northeast rice area of China, where resistance monitoring data are scarce. The results showed no strong *L*. *striatellus* resistance to chlorpyrifos, a broad-spectrum organophosphate used to control *L*. *striatellus*, with RRs ranging from 2.27 to 9.52 in seven field *L*. *Striatellus* populations, while Ban *et al*. (2012) reported chlorpyrifos RRs of 10–61.8 with 11 (out of 13) *L*. *Striatellus* populations having RRs > 22^6^. This apparent increase in chlorpyrifos susceptibility could be a consequence of the reduced use of chlorpyrifos, which is being rotated with other insecticides, such as thiamethoxam and pymetrozine, to slow down resistance development in field populations. Pymetrozine is a new insecticide with a novel mode of action against sucking insects such as the planthopper. Previous results found that *L*. *Striatellus* was susceptible or had a low level of resistance to pymetrozine (RR = 0.5–6.7) in 18 field populations tested between 2010 and 2013 in China^5,6^. Pymetrozine use has been increasing among Chinese farmers with government subsidy in recent years, and we found that field *L*. *striatellus* had developed a moderate level of resistance to pymetrozine (RR of 18.7–34.5). This result suggests that pymetrozine has been overused, and action is required to slow down the development of resistance.

Buprofezin is an insect growth regulator and has a long history of use in planthopper control. High levels of buprofezin resistance have developed in several pest species^6,26,27^. The current study showed that *L*. *striatellus* in eastern China has developed an alarmingly high resistance level to buprofezin with high RR (108.8–156.1), but in the northeast Changchun population, only moderate resistance to buprofezin with an RR of 26.6 is present. This finding suggests that buprofezin use should be limited in eastern China by rotation with other insecticides. Thiamethoxam is a neonicotinoid insecticide that has been used for planthopper control in rice since the 1990s, and no apparent small brown planthopper resistance to thiamethoxam was observed in this study, in line with Ban *et al*. (2012) and Zhang *et al*. (2014)^5,6^, which suggests that *L*. *striatellus* still maintains its susceptibility to thiamethoxam, which continues to be an effective pesticide for *L*. *striatellus*. Overall, the current study revealed that thiamethoxam and chlorpyrifos could control *L*. *striatellus*. Buprofezin should be avoided in eastern China, and pymetrozine should be rotated with other insecticides.

P450 monooxygenase-mediated detoxification is among the major insecticide resistance mechanisms, involving the altered expression of multiple genes in many insect species^15–17,21^. Our previous study found the expression levels of *LsCYP6CW1* to be associated with buprofezin resistance levels^22^. In this study, in line with the buprofezin resistance levels, we found *LsCYP6CW1* to be uniquely over-expressed in all field populations of *L*. *striatellus*, which further implicates overexpressed *LsCYP6CW1* in field resistance to buprofezin. *LsCYP6CW1* expression could provide an effective molecular marker to detect buprofezin resistance in the field in future tests. Moreover, the overexpression of P450 genes might also be important for cross-resistance among different insecticides^28^. We found that the overexpression of *LsCYP6CW1* was to some extent consistent with field resistance to pymetrozine, so we propose that overexpressed *LsCYP6CW1* might also be important for cross-resistance between buprofezin and pymetrozine in field populations. Other relatively less overexpressed P450 genes, such as *LsCYP304H1V3*, *LsCYP305* and *LsCYP4B*, might play secondary roles in insecticide resistance in field *L*. *striatellus* populations.

Previous studies have reported that the ingestion of double-stranded RNA (dsRNA) can effectively suppress target genes in *L*. *striatellus*, and this ability has been widely used in gene function research^23,24,29^. We further evaluated the resistance function of *LsCYP6CW1* in field populations via RNAi. The ingestion of 0.2 mg/mL of *dsLsCYP6CW1* for 5d resulted in an effective suppression of *LsCYP6CW1* expression and significantly increased susceptibility to both buprofezin and pymetrozine compared to the control in two selected field-resistant populations, where the expression of *LsCYP6CW1* was significantly higher than for the other P450 genes. This result further implicated overexpressed *LsCYP6CW1* was an important factor that conferring cross-resistance to these two insecticides in field *L*. *striatellus* populations. Moreover, the resistance mechanism may be complicated in field populations because of the application background of different insecticides. In this study, we found that overexpressed *CYP6CW1* was involved in buprofezin and pymetrozine resistance in field *L*. *striatellus*, but the regulatory mechanism of overexpressed *CYP6CW1* in the field is unclear. One situation that could occur in the field populations of *L*. *striatellus* is that the overexpressed *CYP6CW1* might be regulated by other insecticide selection pressures but not correlated or weakly correlated with the application of buprofezin and pymetrozine, however, as *CYP6CW1* could detoxify both buprofezin and pymetrozine, further studies on metabolism of buprofezin and pymetrozine by P450 enzymes in field populations of *L*. *striatellus* could throw light on the association of overexpression of *CYP6CW1* vis a vis metabolism of these insecticides.

Although the results of this study suggest that *LsCYP6CW1* is involved in cross-resistance to both buprofezin and pymetrozine in field *L*. *striatellus*, our work showed that piperonyl butoxide (PBO) had no substantial synergistic effect on buprofezin and pymetrozine resistance in field populations of *L*. *striatellus*. There are several possible explanations for the results. 1) A number of P450 enzymes are associated with detoxification of a different groups of insecticides, there may be a possibility the PBO has more affinity for other P450 enzymes than the products of *LsCYP6CW1*. 2) Rice planthoppers are plant-sucking pests, and whether or not the rice-stem dipping bioassays used in the current study provided an effective systemic delivery of PBO to the rice seedlings deserves further exploration. 3) A previous study also found that the PBO synergist could decrease the activation of a proinsecticide, which indicated that PBO might be a poor inhibitor of the P450(s) responsible for resistance to some insecticides^30^, and any resistance suppression in this study could be misleading.

## Methods

### Insects

The susceptible (YN) population of *L*. *striatellus*, which contained thousands of individuals, was collected from the Yunnan province of China in July 2001 and has been reared without insecticides since that time. Seven field populations of *L*. *striatellus* were collected in 2015: Nanjing (NJ), Liyang (LY), Jianhu (JH) and Jiangyan (JY) of Jiangsu, Changchun (CC) of Jilin, Jiaxing (JX) of Zhejiang and Lujiang (LJ) of Anhui province. The insects were all reared on rice seedlings at 27 ± 1 °Cunder a 14:10 h light:dark cycle. Field collected insects were mass mated. The third-instar nymphs of F1 or F2 progeny were used for bioassays.

### Bioassays

The degrees of resistance of the NJ, LY, JH, JY, CC, JX, LJ and YN populations against chlorpyrifos, pymetrozine, buprofezin and thiamethoxam were assayed by the rice seedling dip bioassay method based on previous approaches^26^. The formulated insecticides were diluted in distilled water to generate six serial dilutions. A group of four rice seedlings was immersed individually into the insecticide dilutions for 20 s. After air-drying, the seedlings were placed in disposable plastic cups with moistened paper to maintain the wetness of the rice seedling roots. Fifteen 3rd-instar nymphs were placed in each treated plastic cup. Rice seedlings dipped in distilled water that contained 1% TritonX-100 were used as controls. For each dilution, three replicates each containing 15 individuals were treated. For analysis of the synergistic effect of the enzyme inhibitors on these four insecticides, 15 mg L^−1^ PBO was added to each dilution. All tests were maintained at 27(±1) °C, and mortality was recorded after 48 h, 120 h, 120 h and 120 h for chlorpyrifos, pymetrozine, buprofezin and thiamethoxam, respectively. Bioassay data were subjected to probit analysis using the Polo Plus software.

### Screening of *L*. *striatellus* P450 genes associated with insecticide resistance

The candidate P450 genes used in this study came from previous research^7,22^. We newly integrated these p450 genes and deleted the short segments. After the candidate P450 genes were obtained, a BlastP search was performed in NCBI for further support of the annotation predictions, and we finally retained 47 P450 gene sequences in *L*. *striatellus*. Total RNA was extracted from 3rd-instar nymphs of the field and YN populations using the SV Total RNA Isolation system (Promega). Three independent RNA preparations were made for each population, each containing 15 individuals. The first-strand cDNA was synthesized from 2 µg of total RNA using an oligo(dT)15 primer and Superscript III reverse transcriptase (Promega). The relative expression levels of the 47 P450 genes in the YN and field populations were determined by qPCR with ADP ribosylation factor (ARF) as a reference^31^. The PCR primer sequences and the expected size of each PCR product are shown in a previous study and in Table 2 ^31^. The qPCR was performed on three biological samples for each *L*. *striatellus* population, and each sample was analysed in three technical replicates on an Applied Biosystems 7500 thermocycler. The PCR mixture contained 10 µL of SYBR Premix Ex Taq™ (Takara, Japan), 1 µL of cDNA, 0.4 µL of ROX Reference Dye (50×), and 0.4 µL of 10 µM sense and antisense primers in a total volume of 20 µL. The optimized cycling programme was 1 cycle of 95 °C for 30 s, 40 cycles of 95 °C for 5 s, 60 °C for 31 s, and a final disassociation stage that was automatically added by the 7500 System SDS software. The relative expression was calculated using the 2^−ΔΔCT^method^32^. Student’s t-test was performed to determine whether the differences in the relative expression of the 47 P450 genes in the field and YN populations were significant.

**Table 2:** The added primer sequences used in qPCR and dsRNA synthesis.

**Gene**	**Primer sequence (5′ –3′)**	**length(bp)**
*LsCYP425B1*	F:GAAACATCAAAGGGACTGAAGGAG	194
	R: GCCCAACAGAACCAGCACATA	
*LsCYP302A1v2*	F: CTGGCTATTTCTTGGGAGCAATCC	249
	R: TCCTCAACGGTACAGTCACATGGTG	
*LsCYP4DJ1*	F: GCCTCATCCCTCAGTTGCTCGTC	207
	R: CCGCTTCACCGACTCAGTTATCCA	
*LsCYP6CS2v1*	F: TTCTTTGGACGGCGAGGAGT	218
	R: TGCCTATGGCGGTCTTAGCA	
*LsCYP306A1*	F: AGTCAAATACAAATCCGCCCGTCC	278
	R: CACTGCTGCTACCCTGGTTGCTCTA	
*LsCYP307A1v2*	F: CACCGTCCACATCAAGCCACT	218
	R: TCCATCCCGCCAACTTTTCC	
*LsCYP439A1v3*	F: CCTGGAGTTATCCATGTCCATTTC	218
	R: GGACTTCCTTGATGACTCTTTCG	
*LsCYP4DE1*	F: AAAGGCGTTTGAGGAGTTGG	278
	R: GTAGAAAATTGTCGGGGTCGA	
*LsCYP6FU1*	F: GCGTCTTTAGCAACCCTCCC	246
	R: GATCGTGCTGTTCGTTCGTGT	
*LsCYP6CW1*	R: ***TAATACGACTCACTATAGGG***TGCTGAACGAAACCCTGAGAC	372
	F: ***TAATACGACTCACTATAGGG***GCTGTACAATTGAATCGCTCAT	
*GFP-T7*	R:***TAATACGACTCACTATAGGG***GGCGAAGTTCAGCGTGTCCG	426
	F: ***TAATACGACTCACTATAGGG***GGCGCACCTTGATGCCGTTC	

Bold italic indicates the T7 RNA polymerase promoter sequences.

### Resistance functional analysis through RNA interference (RNAi)

A 372 bp segment of the *LsCYP6CW1* cDNA and a 426 bp fragment of the green fluorescent protein (GFP) gene as a control were amplified by PCR. Both PCR products were individually sub-cloned into the pGEM-T easy vector (Promega), and the diluted plasmids were used as templates for the amplification of these target sequences with specific primers that were combined with the T7 RNA polymerase promoter (Table 2). The PCR products were purified with Wizard® SV Gel (Promega) and used as templates for dsRNA synthesis with the T7 Ribomax TM Express RNAi System, according to the manufacturer’s instructions (Promega). The dsRNAs of *LsCYP6CW1* and *GFP* were dissolved in ultrapure water, and the quality and concentrations of the dsRNAs were determined by agarose gel electrophoresis and a Nanodrop 2000 spectrophotometer (Thermo Scientific, Wilmington, DE, USA). Forty 1st-instar nymphs were carefully transferred into the chamber of a glass cylinder (12 cm in length and 2.8 cm in internal diameter) with a liquid artificial diet between two layers of stretched Parafilm^®^ M (Pechiney Plastic Pack ageing Company, Chicago, IL, USA); the stretched Parafilm^®^ M was placed at one end of the chamber, and dense breathable gauze was placed at the other end. The diet was changed and dead nymphs removed daily. Six independent preparations were made for the 200 mg/mL ingestion treatment. Forty-five insects were collected 5 d after ingestion to determine the expression levels of *LsCYP6CW1* using qPCR. A group of insects not fed dsRNA were also tested. Furthermore, we selected two populations in which the expression fold of *LsCYP6CW1* was significantly higher than that of other P450 genes of *L*. *striatellus* to evaluate the resistance role of *LsCYP6CW1* between pymetrozine and buprofezin in the field populations. Changes in the susceptibility of the JY and JX populations against buprofezin and pymetrozine after RNAi were evaluated using the rice seedling dipping methods as mentioned above.
